# Iodide of Calcium as an Antiseptic

**Published:** 1890-06-21

**Authors:** 


### IODATE OF CALCIUM AS AN ANTISEPTIC.

This reagent, which has recently been brought forward as a novelty, was recognized as an antiseptic and disinfectant eighteen years ago by Mr. Sonstadt, who has kept urine and boiled meat perfectly free from odour, and apparently fresh ever since. It is not well suited for surgical purposes, requiring 400 parts of water for its solution, and being not more powerful than boric acid, as well as slow in action. But its non-irritating character, together with its elimination by the kidneys, render it most suitable in chronic cystitis and nephritic abscess, as well as for washing out the bladder in the former cases. It renders and maintains the urine clear and inoffensive.

